# The Set of Fear Inducing Pictures (SFIP): Development and validation in fearful and nonfearful individuals

**DOI:** 10.3758/s13428-016-0797-y

**Published:** 2016-09-09

**Authors:** Jarosław M. Michałowski, Dawid Droździel, Jacek Matuszewski, Wojtek Koziejowski, Katarzyna Jednoróg, Artur Marchewka

**Affiliations:** 1 Faculty of Psychology, University of Warsaw, Warsaw, Poland; 2 Laboratory of Brain Imaging, Neurobiology Centre, Nencki Institute of Experimental Biology, Polish Academy of Sciences, Warsaw, Poland; 3 Laboratory of Psychophysiology, Department of Neurophysiology, Nencki Institute of Experimental Biology, Polish Academy of Sciences, Warsaw, Poland

**Keywords:** Pictures, Visual stimuli, Affective ratings, Valence, Arousal, Fear, Phobia, Emotion

## Abstract

Emotionally charged pictorial materials are frequently used in phobia research, but no existing standardized picture database is dedicated to the study of different phobias. The present work describes the results of two independent studies through which we sought to develop and validate this type of database—a Set of Fear Inducing Pictures (SFIP). In Study 1, 270 fear-relevant and 130 neutral stimuli were rated for fear, arousal, and valence by four groups of participants; small-animal (*N* = 34), blood/injection (*N* = 26), social-fearful (*N* = 35), and nonfearful participants (*N* = 22). The results from Study 1 were employed to develop the final version of the SFIP, which includes fear-relevant images of social exposure (*N* = 40), blood/injection (*N* = 80), spiders/bugs (*N* = 80), and angry faces (*N* = 30), as well as 726 neutral photographs. In Study 2, we aimed to validate the SFIP in a sample of spider, blood/injection, social-fearful, and control individuals (*N* = 66). The fear-relevant images were rated as being more unpleasant and led to greater fear and arousal in fearful than in nonfearful individuals. The fear images differentiated between the three fear groups in the expected directions. Overall, the present findings provide evidence for the high validity of the SFIP and confirm that the set may be successfully used in phobia research.

Anxiety disorders are among the most common emotional disorders in children and adults (Bernstein, Borchardt, & Perwien, 1986; Cartwright-Hatton, McNicol, & Doubleday, 2006). People suffering from severe anxiety experience a substantial psychosocial impairment and refrain from participation in various life activities or interpersonal interactions. In the last several years, the advance of behavioral and neuroimaging methods has allowed us to make considerable progress in understanding the cognitive and neurobiological mechanisms underlying the development, maintenance, and successful treatment of anxiety disorders. Anxiety research has primarily focused on social phobia (also known as *social anxiety disorder*) as well as on the small-animal and blood–injection–injury subtypes of specific phobia that were considered as model disorders for exploring the neurobiological and cognitive mechanisms of anxiety. Most previous findings were obtained using experimental procedures that involved exposure to emotionally charged lexical and pictorial materials. The latter were suggested to have a more direct access to the defensive motivational circuits than do linguistic stimuli (Lang, 1984, 1994). Moreover, previous functional resonance imaging (fMRI) and event-related potential (ERP) studies indicated that emotional pictorial stimuli are processed with shorter latencies (Hinojosa, Carretié, Valcárcel, Méndez-Bértolo, & Pozo, 2009; Schacht & Sommer, 2009) and trigger stronger and more widespread activations than emotional words (Kensinger & Schacter, 2006).

Currently, the pictorial materials employed in anxiety research originate usually from existing standardized affective stimulus databases. Most small-animal and blood–injection phobia studies have included pictures from the International Affective Picture System (IAPS; Lang, Bradley, & Cuthbert, 2008), Geneva Affective Picture Database (GAPED; Dan-Glauser & Scherer, 2011), or Emotional Picture System (EmoPics; Wessa et al., 2010). Individuals suffering from social phobia have mostly been exposed to pictures of faces taken from the Karolinska Directed Emotional Faces (Lundqvist, Flykt, & Öhman, 1998), Japanese and Caucasian Facial Expressions of Emotion (Matsumoto & Ekman, 1988), Montreal Set of Facial Displays of Emotion (Beaupré, Cheung, & Hess, 2000), and NimStim (Tottenham et al., 2009). However, the use of most databases is limited, due to an insufficient number of pictures required to study phobic individuals. This seems to be fairly problematic if one needs to include a great number of stimuli—for instance, when studying ERPs or differences between brain responses to “new” and “old” stimuli (Michalowski, Pané-Farré, Löw, Weymar, & Hamm, 2012; Michalowski, Weymar, & Hamm, 2014; Nowicka, Marchewka, Jednoróg, Tacikowski, & Brechmann, 2010). Also, the picture quality is not always satisfactory when compared to the typical resolution of digital photography at present. For example, the GAPED and EmoPics databases include images with relatively low resolutions of 640 × 480 and 800 × 600 pixels, respectively. Finally, to the best of our knowledge, the existing affective databases lack normative ratings of fear collected in specific representative groups of fearful individuals.

Addressing these issues, Marchewka, Żurawski, Jednoróg, and Grabowska (2014) developed a new standardized database of emotionally salient and neutral pictures—the Nencki Affective Picture System (NAPS). The NAPS database allows researchers to select a variety of different pictures from a pool of 1,356 photographs that have been divided into five categories—people, faces, animal, objects, and landscapes. In addition, pictures have been rated in line with dimensional and categorical models of affect (Riegel et al., 2016). Stimuli were also analyzed for their physical properties, including luminance, contrast, and entropy. However, as in the other previously described databases, the NAPS database has multiple constraints that reduce its usability for anxiety research. First, it does not include a sufficient number of fear-relevant and specific-phobia-related images, necessary for studying the full spectrum of fear disorders. Second, the normative ratings that are available for the NAPS pictures were collected only in healthy populations. Due to the limitations of the existing resources, anxiety researchers have employed stimuli not only from available affective databases, but also from public (e.g., Internet) or private collections. Therefore, it is difficult to control the ecological validity and physical characteristics of these stimulus materials and compare quantitatively the results obtained across different studies.

The present work describes the development of a standardized set of photographs for measuring different phobic disorders—the Set of Fear Inducing Pictures (SFIP). First, we selected and divided photographs into five categories: social exposure, blood/injection, small animals, angry faces, and neutral images. Pictures of angry faces are known to elicit fear in social phobics (see Brühl, Delsignore, Komossa, & Weidt, 2014, for a review) and were included here to validate the fear-inducing properties of the new social-exposure picture category. Pictures were taken from the IAPS (Lang et al., 2008), GAPED (Dan-Glauser & Scherer, 2011), NAPS (Marchewka et al., 2014), and the Warsaw Set of Emotional Facial Expression Pictures (WSEFEP; Olszanowski et al., 2015), as well as from freely available nonprofit photography stocks or pictures taken by the coauthors. Two studies were performed to develop and validate the SFIP. In Study 1, normative ratings of valence, arousal, and fear were collected for each picture from small-animal fear, blood/injection fear, and social fear groups, and from a nonfearful control group. Here, participants were selected on the basis of scores from the small-animal, blood/injection, and social/failure subscales of the Fear Survey Schedule (FSS-III; Wolpe & Lang, 1964). The Study 1 ratings were used to select pictures for the final version of the SFIP, which was later evaluated in Study 2 by spider fear, blood/injection fear, social fear, and control groups selected on the basis of three phobia-specific questionnaires. The NAPS images (*N* = 753) as well as pictures from freely available non profit photography stocks and those taken by the authors (*N* = 134) that are included in the SFIP are free to download after filling out the official form, available from the following public webpage: http://naps.nencki.gov.pl/. This webpage also provides access to the SFIP picture ratings and their physical features (luminance, contrast, and complexity). The stimuli from IAPS, WSEFES, and GAPED are available from the original authors.

## Study 1: The development of the SFIP

In Study 1, we aimed to construct the SFIP by selecting photographs that were fear- and/or arousal-inducing in people who suffer from social, small-animal, or blood/injection fear. The construction of the SFIP was done by balancing two goals: The photographs had to trigger increased fear and/or arousal in one of the three fear groups, while at the same time eliciting less intense fear and/or arousal in the other fearful and nonfearful individuals.

### Method

#### Participants

Our participants were recruited from a large sample of students from Warsaw universities (*N* = 1,671) who completed an online version of the 92-item FSS-III (Wolpe & Lang, 1964). Participants were asked to evaluate their subjective experience of fear for each FSS item on a 5-point Likert-type scale ranging from 1 (*not at all*) to 5 (*very much*). Replicating previous findings (for a review, see Arrindell et al., 1991), factor analyses showed acceptable fit of the three-factor solution. One factor contained 20 items reflecting social anxiety or fear of failure (e.g., speaking in public, failing, and being teased), the second included 12 items assessing fear of blood/injection/injury (e.g., open wounds, dead people, and receiving injections), and the third included seven items reflecting fear of small animals (e.g., bats, harmless spiders, flying insects, and harmless snakes). Participants scoring greater than or equal to one standard deviation above the mean (≥+1 *SD*) on one of these factors and less than one standard deviation above the mean (<+1 *SD*) on the other two factors were assigned to an appropriate fear group: small-animal fear (SA), blood/injection/injury fear (BII), or social/failure fear (SOC). Those scoring less than or equal to the mean on all three factors were preselected as the nonfearful controls (CON). A group of 117 preselected candidates (26 male [♂], 91 female [♀]) participated in the picture-rating session, including 22 control (14♂, 8♀), 35 social/failure fear (6♂, 29♀), 34 small-animal fear (3♂, 31♀), and 26 blood/injection fear (3♂, 23♀) individuals. Descriptive statistics for the individual FSS subscales and the total scores are presented in Table 1. When compared to the other groups, the small-animal fear group showed significantly higher FSS Small Animal subscale scores, the blood fear group revealed higher FSS Blood/Injection subscale scores, and the social fear group higher FSS Social/Failure subscale scores, *t*s > 10, *p*s < .001. All fear groups scored significantly higher on their fear-specific FSS subscales than on the other scales, *t*s > 7, *p*s < .001. The control group showed significantly higher scores on the FSS Blood and Social/Failure subscales than on the FSS Small Animal subscale, *t*s(21) > 2.5, *p*s < .05, and no differences between the FSS Blood and Small Animal subscales, *t*(21) = 1.58, n.s.

**Table 1:** Mean scores and standard deviations on the Fear Survey Schedule (FSS) in the fear and control samples

	Control	Social/Failure Fear	Small Animal Fear	Blood/Injection Fear
FSS Social/Failure	1.56 (0.53)	3.70 (0.29)	2.62 (0.48)	2.58 (0.41)
FSS Small Animals	1.14 (0.19)	1.90 (0.46)	3.48 (0.50)	2.10 (0.48)
FSS Blood/Injection	1.37 (0.36)	2.04 (0.57)	2.40 (0.49)	3.58 (0.34)
FSS total score	1.35 (0.26)	2.37 (0.28)	2.25 (0.27)	2.35 (0.20)

The scores range from 1 to 5.

#### Materials

**Fig. 1:**
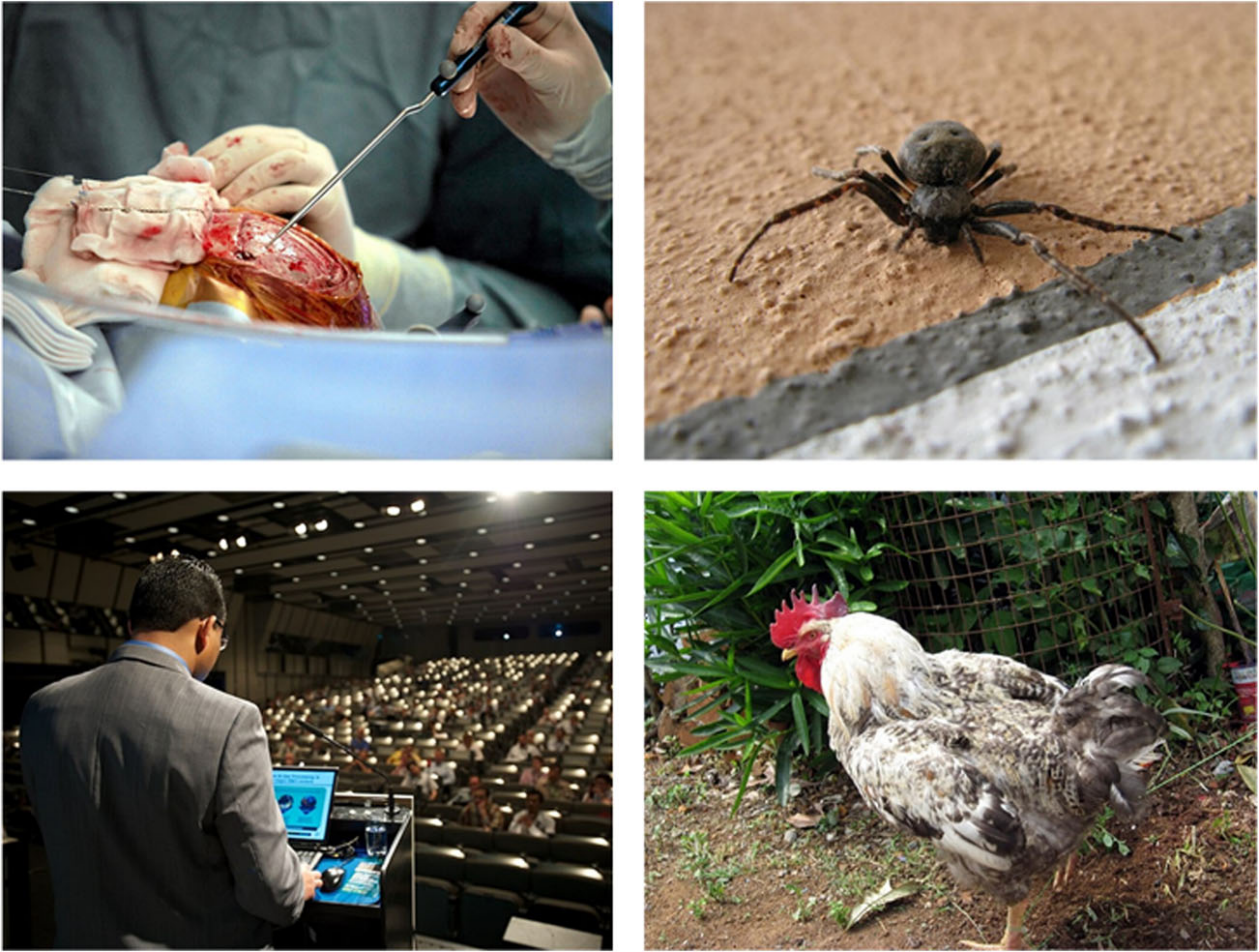
Examples of photo categories. From top-left to bottom-right: blood/injection phobia, spider phobia, social phobia, neutral

Participants were presented with a total of 400 color pictures. 151 photographs were taken from Flickr (https://www.flickr.com/) under a Creative Commons license (https://www.flickr.com/creativecommons/) or taken by the coauthors. In addition, 12 images were taken from the IAPS (Lang et al., 2008), seven images from GAPED (Dan-Glauser & Scherer, 2011), 170 images from NAPS (Marchewka et al., 2014; Riegel et al., 2016; Wierzba et al., 2015), and 60 images from WSEFEP (Olszanowski et al., 2015). The selected pictures could be divided into five categories: blood/injection (*N* = 80 from NAPS), small animals (*N* = 80, including seven GAPED and four IAPS), social exposure (*N* = 80, including 18 NAPS and four IAPS), angry faces (*N* = 30 from WSEFEP), and neutral (*N* = 130, including 74 NAPS, 30 WSEFEP, and four IAPS; see Fig. 1). A picture was selected for the social-exposure, blood/injection, or small-animal category if its content referred to the items of the corresponding FSS subscale. The social-exposure category included photographs illustrating items from the FSS Social/Failure subscale (e.g., speaking in public, being teased, or taking an exam). The blood/injection category consisted of photographs depicting the FSS Blood/Injection subscale’s items (e.g., open wounds, dead people, or receiving injections), and the small-animal picture category included the FSS Small Animal subscale’s objects (e.g., bats, spiders, flying insects, or snakes). The neutral category included pictures of animals, objects, people, faces, plants, and landscapes. The images were resized and cropped using proportions of 4:3 (portrait) or 3:4 (landscape). All stimuli were divided into two equal sets of 200 images balanced by content. Set 1 was evaluated by 61, and Set 2 by 56, participants.

#### Procedure

Prior to the computerized experimental procedure that was run in the laboratory, participants were familiarized with examples of the images, and asked to provide written informed consent. Each participant judged 200 pictures presented pseudorandomly with the restriction that pictures from the same category could not occur on three consecutive trials. During the rating session each picture was presented for 3 s in the middle of the 21,5-in. monitor covering approximately 65 % of the screen space. After the offset of the slide, the smaller version of the picture remained available on the top of the screen until it was evaluated on all of the three scales: fear, arousal, and valence. First, the participant was asked to evaluate his/her subjective experience of fear on a scale ranging from 1 (*not at all*) to 5 (*very much*). Following the fear decision, each participant rated his or her subjective experience of valence (displeasure–pleasure) and arousal on a computerized version of the 9-point Likert scale of the Self-Assessment Manikin—a popular pictorial self-report technique used to directly assess the intensity and the direction of affective reactions to various kinds of stimuli (Bradley & Lang, 1994). The arousal scale ranged from 1 (*calm*) to 9 (*aroused*), and the valence scale ranged from 1 (*unpleasant*) to 9 (*pleasant*). The rating session lasted approximately 40 min and was conducted on standard PC computers with 21.5-in. LCD computer monitors. Stimulus presentation and data acquisition were based on in-house software. All responses were analyzed using the SPSS package. The study protocol was approved by the local ethics committee and conformed to Standard 8 of the American Psychological Association’s Ethical Principles for Psychologists and Code of Conduct.

#### SFIP construction and statistical methods

From the initial 400 photographs, 288 pictures were selected for the SFIP on the basis of their content validity. An image was included into one of the three phobia-subsets if it was rated as more fear- and/or arousal-evoking by the corresponding fear group than by the remaining groups. Considering these criteria, 172 fear-relevant pictures were selected: 80 blood/injection (alive injured or dead human or animal bodies, or their isolated parts), 40 social-exposure scenes (microphones, people sweating, taking an exam, or giving a lecture), 30 angry faces, and 22 small-animal pictures (i.e., spiders and bugs). Since the ratings in the latter category were characterized by high variance and some subcategories of the small animals did not discriminate between the small-animal fear and the other fear groups in the expected direction, in the final SFIP version we included only photographs depicting spiders and bugs. The picture pool was completed with 116 neutral photographs (e.g., objects, people, mushrooms, plants, landscapes, animals, and 30 neutral faces) that did not evoke fear in any of the four experimental groups (i.e., fear *M* < 2.0, or less than *a little*). The initial set of 400 pictures and the resulting set of 288 pictures selected to the SFIP were analyzed separately. In each case, independent group *t* test, means, standard deviations, and Cohen’s *d* are reported in the Results section to describe the between-group effects separately for each picture category.

### Results

#### Fear ratings

**Fig. 2:**
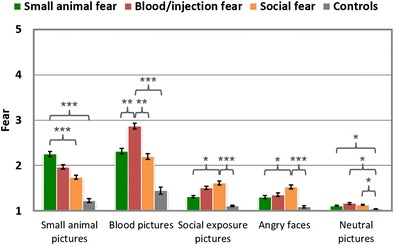
Mean fear ratings of different phobic groups in response to specific stimuli categories. Error bars represent *SEM*s. ^***^ *p* < .001; ^**^ *p* < .01; ^*^ *p* < .05

All fear-relevant picture categories obtained higher fear ratings in the corresponding fear groups than among the nonfearful controls, *t*s ≥ 3.6, *p*s < .001 (see Table 2 and Fig. 2). Here, the effect size was largest for the blood pictures (BII > CON: *d* = 2.10), followed by photographs of small animals (SA > CON: *d* = 1.77), social exposure (SOC > CON: *d* = 1.39), and angry faces (SOC > CON: *d* = 1.09). The evaluation of neutral pictures revealed higher fear ratings in fearful than in control individuals, *t*s > 2.4, *p*s < .05, effect sizes ranging from 0.63 (SA > CON) to 0.82 (BII > CON). Comparing the three fear groups revealed that higher fear ratings were found for small-animal pictures in the SA than in the SOC and BII groups, *t*(67) = 3.52, *p* < .001, *d* = 0.84, and *t*(58) = 1.80, *p* = .078, *d* = 0.45, respectively. Blood/injection pictures were rated as being more fear-evoking by the BII than by the SOC and SA groups, *t*s > 3, *p*s < .01 (*d*s = 0.94 [BII > SOC] and 0.79 [BII > SA]). Social-fearful participants rated pictures of social exposure and angry faces as being more fear-evoking than did the SA group, *t*s > 2, *p*s < .05, moderate effect sizes (i.e., between 0.5 and 0.7), but not than the BII group, *t*s(59) < 1.5, *p*s > .1.

**Table 2:** Mean fear, arousal, and valence ratings (and standard deviations), displayed as a function of group for each picture category

Category	Control			Social/Failure Fear			Small Animal Fear			Blood/Injection Fear		
	Fear	Arousal	Valence	Fear	Arousal	Valence	Fear	Arousal	Valence	Fear	Arousal	Valence
Neutral	1.04 (0.12)	1.57 (0.88)	6.14 (0.93)	1.13 (0.13)	2.21 (0.99)	5.91 (0.52)	1.11 (0.10)	1.77 (0.90)	6.01 (0.87)	1.16 (0.17)	2.32 (1.19)	5.96 (0.73)
Blood/ injection	1.44 (0.71)	2.82 (1.62)	3.69 (1.08)	2.2 (0.76)	4.95 (1.68)	2.88 (0.88)	2.31 (0.75)	4.42 (1.56)	2.68 (0.99)	2.86 (0.64)	5.45 (1.27)	2.26 (0.55)
Angry faces	1.09 (0.22)	1.74 (0.99)	4.83 (0.73)	1.53 (0.54)	3.41 (1.80)	3.92 (0.80)	1.3 (0.37)	2.3 (1.20)	4.39 (1.20)	1.36 (0.42)	2.57 (1.45)	4.34 (1.18)
Social (*N* = 80)	1.1 (0.14)	2.02 (1.01)	5.28 (0.86)	1.61 (0.50)	3.59 (1.53)	4.3 (0.66)	1.32 (0.29)	2.46 (1.22)	4.91 (0.95)	1.51 (0.38)	3.26 (1.45)	4.72 (0.91)
Small animals (*N* = 80)	1.23 (0.43)	2.07 (1.23)	5.5 (1.31)	1.74 (0.51)	3.62 (1.67)	4.48 (0.98)	2.25 (0.69)	3.92 (1.58)	3.52 (1.33)	1.98 (0.48)	3.76 (1.31)	4.13 (0.83)
Social (*N* = 40)	1.09 (0.17)	1.97 (1.04)	5.32 (0.89)	1.75 (0.62)	3.64 (1.67)	4.08 (0.78)	1.34 (0.31)	2.39 (1.23)	4.93 (0.97)	1.53 (0.46)	3.3 (1.53)	4.69 (0.89)
Spiders/bugs (*N* = 22)	1.29 (0.41)	2.19 (1.34)	4.84 (1.48)	2.01 (0.73)	4.04 (1.99)	3.7 (1.14)	2.72 (0.95)	4.56 (1.88)	2.64 (1.49)	2.21 (0.56)	4.16 (1.40)	3.44 (0.76)

#### Arousal ratings

**Fig. 3:**
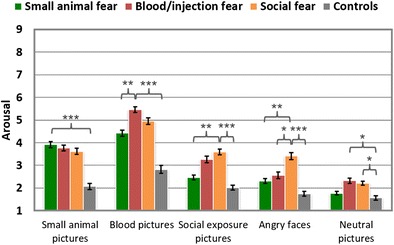
Mean arousal assessments of different phobic groups in response to specific stimuli categories. Error bars represent *SEM*s. ^***^ *p* < .001; ^**^ *p* < .01; ^*^ *p* < .05

Mirroring the fear ratings, arousal assessments for the fear-relevant pictures were higher in the corresponding fear groups than in the control participants, *t*s ≥ 4.0, *p*s < .001 (see Table 2 and Fig. 3). The effect sizes were largest for the blood/injection picture category (BII > CON: *d* = 1.81), followed by pictures depicting small animals (SA > CON: *d* = 1.31), social-exposure scenes (SOC > CON: *d* = 1.21), and angry faces (SOC > CON: *d* = 1.15). When compared to the nonfearful controls, even the neutral pictures were rated as being more arousing in the SOC and BII groups, *t*s > 2.5, *p*s < .05, moderate effect sizes, but not in the SA group, *t*(54) < 1, *p* > .1. For photographs of small animals, arousal was rated similarly in all fear groups, *t*s < 1, *p*s > .1. Blood/injection pictures were rated as being more arousing in the BII than in the SA group, *t*(58) = 2.75, *p* < .01, *d* = 0.72, but not than in the SOC group, *t*(59) = 1.28, *p* > .1. Pictures of social exposure and angry faces were rated as being more arousing in the SOC than in the SA group, *t*s > 3, *p*s < .01 (*d*s = 0.73 and 0.82, respectively). The arousal ratings were higher in the SOC than in the BII group for pictures of angry faces, *t*(59) = 1.96, *p* = .05, *d* = 0.51, but not for social-exposure scenes, *t*(59) < 1, *p* > .1.

#### Valence ratings

**Fig. 4:**
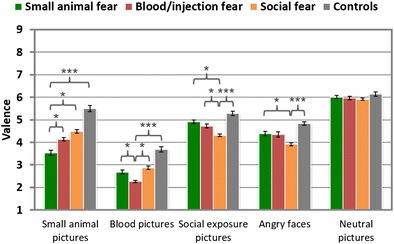
Mean valence ratings of different phobic groups in response to specific stimuli categories. Error bars represent *SEM*s. ^***^ *p* < .001; ^**^ *p* < .01; ^*^ *p* < .05

The three fear groups evaluated their target pictures as being more unpleasant than did the controls, *t*s > 4, *p*s < .001: blood photographs (BII > CON: *d* = 1.67), small-animal pictures (SA > CON: *d* = 1.50), social-exposure scenes (SOC > CON: *d* = 1.28), and angry faces (SOC > CON: *d* = 1.12; see Table 2 and Fig. 4). Neutral pictures were assessed with similar valence ratings by the fear and nonfearful control groups, *t*s < 1.2, *p*s > .1. Photographs of small animals were experienced as being more unpleasant in the SA group than in the SOC and BII groups, *t*s > 2, *p*s < .05 (*d*s = 0.82 and 0.55, respectively). Blood/injection pictures were assessed as being more unpleasant in the BII than in the other fear groups, *t*s ≥ 2, *p*s ≤ .05 (*d*s = 0.84 [BII > SOC] and [BII > SA]). Pictures of social exposure were rated as being more unpleasant in the SOC than in the SA and BII groups, *t*s ≥ 2, *p*s < .05, moderate effect sizes. Images of angry faces were evaluated as being more unpleasant in the SOC than in the SA group, *t*(67) = 2.02, *p* < .05, *d* = 0.75, but not than in the BII group, *t*(59) = 1.66, *p* = .10.

#### Ratings for selected social-exposure pictures

Selecting the social-exposure pictures that best discriminated between the social fear and the other fear groups increased their discrimination abilities. The selected subset was rated as being more fear-evoking, arousing, and unpleasant by social fearful than by nonfearful and small-animal fearful participants, *t*s(55) > 4.4, *p*s < .001, *d*s ≥ 1.2, and *t*s(67) > 3.5, *p*s < .001, *d*s > 0.8, respectively. When compared to the BII group, social-fearful participants rated this set as being more unpleasant, *t*(59) = 2.75, *p* < .01, *d* = 0.73, but similarly arousing and fear-evoking, *t*s(59) < 1.6, *p*s > .1 (see Table 2, bottom). In the social fear group, this set of social-exposure pictures was rated as being more arousing and fear-evoking than pictures depicting angry faces, *t*(35) = 2.4, *p* < .05, and *t*(35) = 2.0, *p* = .056, for fear and arousal ratings, respectively.

#### *Ratings for pictures of spiders and bugs*^1^

The pictures of spiders and bugs selected from the small-animal set (see the Discussion below) differentiated the SA from the other groups. Here, SA participants rated these pictures as being more fear-evoking, arousing, and unpleasant than did the nonfearful controls, *t*s(54) > 5, *p*s < .001, ds > 0.9. As compared to the SOC and BII groups, the SA group found these pictures more unpleasant, *t*s > 2.5, *p*s < .05 (*d*s = 0.80 [SA > SOC] and 0.68 [SA > BII]), and fear-evoking, *t*s ≥ 2.5, *p*s < .05 (*d*s = 0.84 [SA > SOC] and 0.65 [SA > BII]), but similarly arousing, *t*s < 1.2, *p*s > .1.

### Discussion

In Study 1, we selected and validated a set of stimuli for studying fear responses in social and specific (blood and spider) fearful individuals. The results of this study revealed that pictures depicting small animal, as well as social-exposure and blood/injection scene, were assessed as being more fear-evoking, arousing, and unpleasant by the corresponding fear groups than by nonfearful controls. Interestingly, neutral pictures were also assessed as being more arousing and fear-evoking in the social and blood fear groups, suggesting that in a fear-relevant context these participants might tend to assess all of the stimulus materials as being more arousing and fear-evoking, a tendency that might have affected the results of our validation study. As expected, blood/injection pictures were rated as being more unpleasant and fear-evoking in the blood/injection than in the other fear groups. Fear and valence (but not arousal) ratings for small-animal pictures were also shown to differentiate between the small-animal and the other fear groups. Pictures depicting social-exposure scenes were rated as being more arousing, fear-evoking, and unpleasant in the social than in the small-animal fearful participants. At the same time, the social and blood/injection fear groups did not differ in their arousal and fear ratings for social-exposure pictures.

Overall, our findings revealed several weaknesses of the initially selected picture set. First, the subset of small-animal pictures was assessed as being similarly arousing by the three fear groups, and a thorough examination revealed that the group effects were unstable across different subcategories of small animals, suggesting that small-animal phobia cannot be treated homogeneously. As a consequence, we decided that the resulting picture set would only include the pictures depicting spiders and bugs that were shown to best discriminate between the small-animal and the other fear groups (see Table 2). Second, we found no difference between the social and BII fear groups with regard to their arousal and fear ratings for social-exposure pictures. This was mostly related to the fact that the BII individuals were particularly sensitive to those social-exposure photographs that depicted some forms of violence (e.g., being teased or one person bullying another). In fact, previous findings had revealed that BII fearful people avoid violent movies/games due to their association with blood and injuries (Ritz & Meuert, 2014). However, selecting only social-exposure pictures that were assessed with greater fear and/or arousal ratings by social-fearful participants did not meaningfully improve the pictures’ ability to discriminate between the social and blood/injection fear groups. Nonetheless, in the social fear group the selected set of social-exposure pictures was assessed as being more arousing and fear-evoking than the pictures depicting angry faces. Third, the ratings demonstrated that the three fear groups were emotionally more affected by neutral photographs than were the nonfearful controls. Considering the fact that threat was more likely to occur for fearful participants in this study, we suggest that this effect may reflect the greater overall arousal experienced by the fear groups and might be reduced by including a greater number of neutral pictures.

## Study 2: The validation of the SFIP

A second study was performed to improve the SFIP in two ways. First, addressing the weaknesses mentioned above, we aimed to extend the original picture set used in Study 1 by including additional neutral and spider-phobia-relevant pictures, and because the picture set was changed, we intended to collect subjective ratings for the final version of the SFIP. The new set consisted of fear-relevant pictures including 80 original blood/injection pictures, 30 original pictures of angry faces, 40 original social-exposure pictures, and 22 original and 58 new pictures of spiders and bugs. As neutral pictures, we included 116 original and 610 new pictures. We decided to increase the number of neutral pictures about six times so as to reduce the hypothesized tendency of phobic participants to overestimate their fear/arousal assessments in an arousing/fear-relevant context. Our second goal was to validate the SFIP using a more clinically valid way of assigning participants to groups. Therefore, in Study 2 participants were selected on the basis of three phobia-specific and psychometrically sound instruments that are often used to recruit blood, social, and spider phobia participants—that is, the Mutilation Questionnaire (MQ; Kleinknecht & Thorndike, 1990), the Anxiety Subscale of the Leibowitz Social Anxiety Scale (LSAS; Liebowitz, 1987), and the Spider Phobia Questionnaire (SPQ; Klorman, Weerts, Hastings, Melamed, & Lang, 1974).

### Method

#### Participants

A total of 66 individuals (47♀, 19♂; mean age = 23.3 years, *SD* = 4.1) participating in this study were selected from Warsaw universities on the basis of their scores from the Polish versions of the MQ (Kleinknecht & Thorndike, 1990), the Anxiety Subscale of the LSAS (Liebowitz, 1987), and the SPQ (Klorman et al., 1974). Participants scoring greater than or equal to one standard deviation above the mean (≥+1 *SD*) on one of the three fear questionnaires and less than one standard deviation above the mean (<+1 *SD*) on the other two questionnaires were selected into an appropriate fear group (see Table 3). A total of 17 participants (16 females) who reported elevated spider fear (SPQ ≥ 16; *M* = 22.4, *SD* = 3.4) were allocated to the spider fear group (SA). Another 17 participants (11 females) who appeared to be afraid of social situations (LSAS anxiety ≥ 39; *M* = 43.3, *SD* = 6.2) were assigned to the social fear group (SOC). A group of 16 participants (14 females) who scored high on the MQ (MQ ≥ 20; *M* = 21.5, *SD* = 1.5) were included in the blood/injection fear group (BII). And a further 16 participants (nine females) scoring below the mean on all scales were classified as nonfearful controls (CON). The proportion of males and females included in the fear groups is broadly representative for the whole population. All three fear groups scored higher on their fear-specific questionnaires than on the other fear scales, *t*s > 5, *p*s < .001. We observed no differences between the fear questionnaire scores in the control group, *t*s (15) < 2, n.s.

**Table 3:** Mean *z* scores and standard deviations from fear-specific questionnaires in the fear and control samples

	Control	Social/Failure	Spider Fear	Blood/Injection
SPQ	–0.58 (0.28)	–0.32 (0.73)	1.41 (0.41)	–0.30 (0.50)
MQ	–0.98 (0.47)	–0.03 (0.66)	–0.32 (0.69)	1.35 (0.25)
LSAS anxiety	–1.10 (0.50)	1.26 (0.44)	–0.15 (0.69)	0.70 (0.49)–
LSAS avoidance	–1.04 (0.54)	1.09 (0.67)	–0.19 (0.81)	0.09 (0.56)

#### Materials

A pool consisting of 956 photographs was used. Of these pictures, 288 were selected from the pool used in Study 1 (see Fig. 1 above). The pool comprised 726 neutral photographs (including objects, people, mushrooms, plants, faces, landscapes, and animals; four IAPS, 670 NAPS, and 30 WSEFEP pictures) and 230 fear-relevant pictures, divided into three categories: 70 social (30 pictures of angry faces from WSEFEP, and 40 social-exposure scenes, including microphones, people sweating, taking an exam, or giving a lecture; one from the IAPS), 80 spider/bug pictures (including three from IAPS, one from GAPED, and three from NAPS), and 80 NAPS blood/injection pictures (including alive injured or dead human or animal bodies, or their isolated parts).

#### Procedure

On arrival at the laboratory, participants were briefed on the experimental procedure and signed an informed consent form. In order not to overload the participants’ cognitive capabilities, the whole set of 956 pictures was divided into two halves, and each participant was asked to view one half including 478 pictures, with 363 neutral photographs and 115 fear-relevant pictures divided into three categories: 35 social (15 pictures of angry faces and 20 social-exposure scenes), 40 pictures of spiders and bugs, and 40 blood/injection pictures. Consecutive presentation of two stimuli from the same fear-relevant category was avoided. Each picture was presented for 3 s in the middle of the monitor covering approximately 65 % of screen space. After the offset of the picture, the participant was asked to evaluate his or her subjective experience of fear on a scale ranging from 1 (*not at all*) to 5 (*very much*), as well as valence and arousal on a computerized version of the Self-Assessment Manikin (Bradley & Lang, 1994). The rating session lasted approximately 75 min. An obligatory 5-min break was taken after half of the stimuli had been assessed, during which participants were asked to leave the experimental room.

#### Statistical analyses

To describe the overall characteristics of the final SFIP version, statistical analyses were carried out separately for fear, arousal, and valence ratings by calculating repeated measures analyses of variance (ANOVAs), including the within-subjects factor Picture Category (spider/bugs vs. social exposure vs. angry faces vs. blood/injection vs. neutral) and the between-subjects factor Group (spider fear vs. blood/injection fear vs. social fear vs. controls). In each case, independent group *t* test, mean, standard deviation, and Cohen’s *d* are reported in the Results section, to describe the between-group effects separately for each picture category. For effects involving repeated measures, Greenhouse–Geisser correction of the degrees of freedom was applied.

### Results

#### Overall SFIP characteristics

**Fig. 5:**
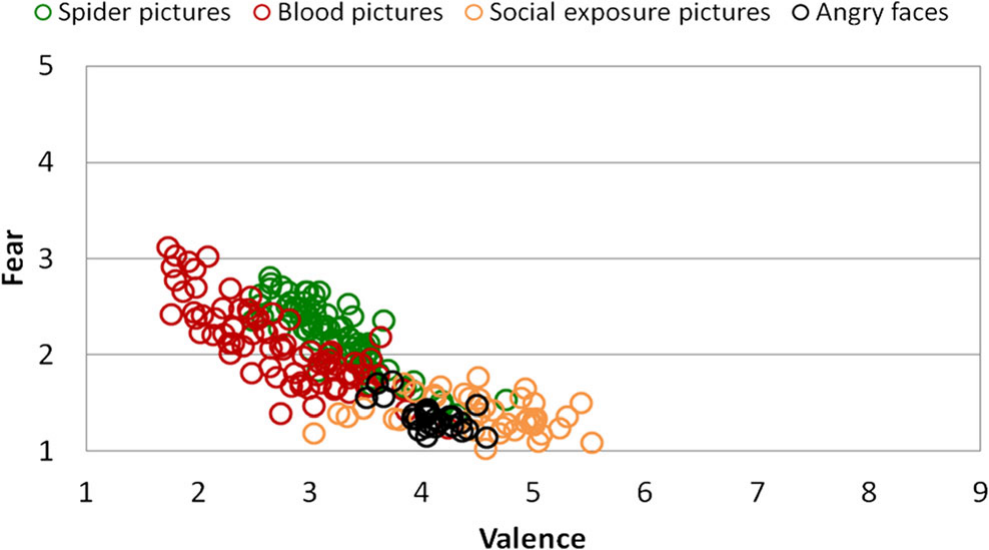
Relationship between fear and valence assessments of all groups in response to pictures from different categories

**Fig. 6:**
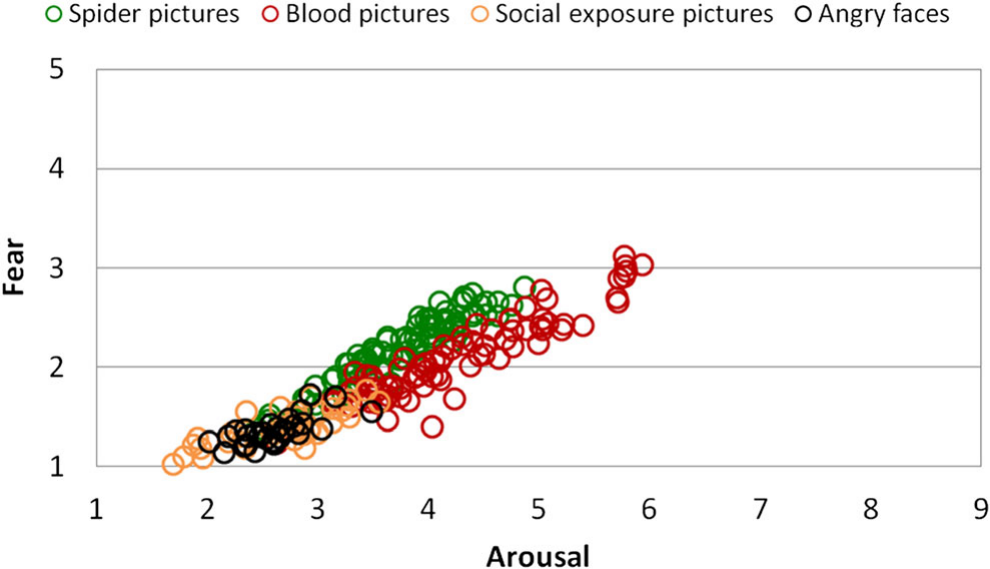
Relationship between fear and arousal ratings of all groups in response to pictures from different categories

Whole-sample overall ANOVAs calculated using the Greenhouse–Geisser correction revealed that the arousal, fear, and valence ratings differed between the single picture categories, *F*s(4, 248) > 100, *p*s < .001, *η*_p_^2^s > .62. Pictures from the blood/injection and spider/bug categories were assessed as being more arousing and fear-evoking, as well as more unpleasant, than the photographs depicting social-exposure scenes and angry faces, *F*s(1, 62) > 53, *p*s < .001, *η*_p_^2^s > .46 (see Figs. 5 and 6). Moreover, when compared to the spider/bug category, blood/injection pictures were rated as being more unpleasant, *F*(1, 62) = 23.67, *p* < .001, *η*_p_^2^ = .28, and arousing, *F*(1, 62) = 10.54, *p* < .01, *η*_p_^2^ = .15, but the two categories were similar on the fear scale, *F*(1, 62) = 1.16, n.s. All fear-relevant pictures were rated as being more arousing, fear-evoking, and unpleasant than the neutral category, *F*s(1, 62) > 53, *p*s < .001, *η*_p_^2^s > .46. Pearson’s correlations calculated to examine the relationship between the ratings of valence, arousal, and fear revealed that all three dimensions were highly correlated, in the case of the fear-relevant picture categories: *r*s(66) > .50, *p*s < .001. For the neutral picture category, only the correlations between fear and arousal ratings were found to be statistically significant, *r*(66) = .66, *p* < .001.

#### Between-group analyses

##### Fear ratings

**Fig. 7:**
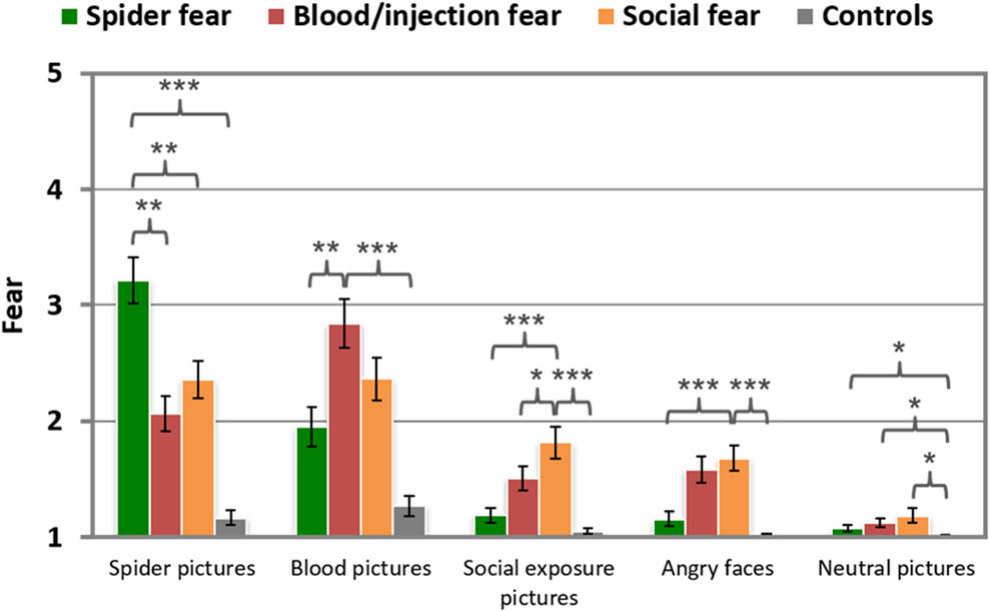
Mean fear ratings of different phobic groups in response to specific stimulus categories. *SEM*s are marked by black lines. ^***^ *p* < .001, ^**^ *p* < .01, ^*^ *p* < .05

Fear-relevant SFIP pictures were rated as being more fear-evoking by the corresponding fear groups than by the controls, *t*s > 5, *p*s < .001 (see Table 4 and Fig. 7). These effects were most strongly pronounced for photographs of spiders/bugs (SA > CON: *d* = 3.33), followed by the blood/injection pictures (BII > CON: *d* = 2.47), angry faces (SOC > CON: *d* = 2.01), and social-exposure scenes (SOC > CON: *d* = 1.84). The evaluation of neutral pictures also revealed higher fear ratings in fearful than in control individuals, *t*s ≥ 2.5, *p*s < .05 (*d*s = 2.14 [BII group], 1.03 [SA group] and 0.92 [SOC group]). The comparisons between the fear groups revealed higher fear ratings for pictures of spiders/bugs in the spider fear group, *t*s > 3.1, *p*s < .01 (*d*s = 1.08 [SA > SOC] and 1.53 [SA > BII]), and for social-exposure pictures in the social fear group, *t*(19,881) = 4.25, *p* < .001, *d* = 1.48 [SOC > SA], and *t* = 1.88, *p* = .070, *d* = 0.66 [SOC > BII]. For blood/injection stimuli fear ratings were higher in the BII than the SA, *t*(31) = 3.28, *p* < .01, *d* = 1.14, but not the SOC group, *t*(30,334) = 1.75, *p* = .091, *d* = 0.60. Angry faces were rated as being more fear-evoking by the social fearful than by the SA group, *t*(32) = 4.02, *p* < .001, *d* = 1.37, but not than by the BII group, *t*(31) < 1, *p* > .1. In the social fear group, pictures of social exposure and angry faces were assessed with similar fear ratings, *t*(16) = 1.70, *p* > .1.

**Table 4:** Mean fear, arousal and valence ratings (and standard deviations) displayed as a function of group for each picture category

Picture Category	Control			Social Fear			Spider Fear			Blood/Injection Fear		
	Fear	Arousal	Valence	Fear	Arousal	Valence	Fear	Arousal	Valence	Fear	Arousal	Valence
Neutral	1.01 (0.02)	1.31 (0.27)	5.58 (0.32)	1.18 (0.26)	2.16 (0.96)	5.59 (0.44)	1.07 (0.08)	1.90 (0.82)	5.56 (0.64)	1.12 (0.07)	2.26 (0.87)	5.79 (0.74)
Blood/ injection	1.26 (0.37)	2.35 (1.06)	3.56 (0.82)	2.37 (0.77)	4.80 (1.69)	2.63 (0.55)	1.95 (0.75)	4.09 (1.54)	2.69 (0.81)	2.85 (0.83)	5.67 (1.07)	2.06 (0.67)
Angry faces	1.02 (0.04)	1.50 (0.59)	4.58 (0.65)	1.69 (0.47)	3.43 (1.34)	3.72 (0.59)	1.17 (0.26)	2.34 (1.34)	4.14 (0.80)	1.58 (0.47)	3.26 (1.04)	3.79 (0.99)
Social	1.05 (0.12)	1.57 (0.70)	4.90 (0.63)	1.82 (0.58)	3.50 (1.59)	3.94 (0.56)	1.18 (0.20)	2.39 (1.24)	4.85 (0.82)	1.49 (0.40)	3.16 (1.21)	4.41 (0.74)
Spiders/bugs	1.16 (0.28)	1.72 (0.84)	4.49 (0.95)	2.39 (0.68)	4.31 (1.82)	2.87 (0.85)	3.20 (0.82)	5.21 (1.63)	2.07 (0.78)	2.04 (0.69)	3.83 (1.43)	3.53 (1.22)

##### Arousal ratings

**Fig. 8:**
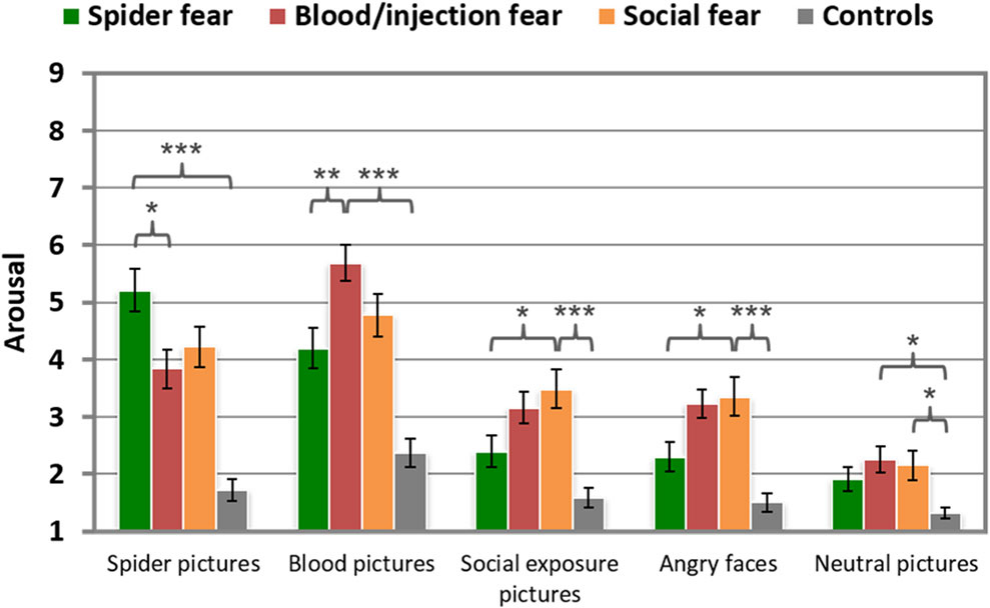
Mean arousal ratings of different phobic groups in response to specific stimulus categories. *SEM*s are marked by black lines. ^***^ *p* < .001, ^**^ *p* < .01, ^*^ *p* < .05

Arousal ratings for the fear-relevant pictures were higher in the corresponding fear groups than in the control participants, *t*s > 4.4, *p*s < .001 (see Table 4 and Fig. 8). Here, the effect sizes were largest for the blood picture category (BII > CON: *d* = 3.12), followed by the pictures of spiders/bugs (SA > CON: *d* = 2.69), angry faces (SOC > CON: *d* = 1.86), and social-exposure scenes (SOC > CON: *d* = 1.57). Greater arousal ratings were observed in fearful than in nonfearful participants also for the neutral pictures, *t*s > 2.7, *p*s < .05 (effect sizes between 0.97, for SA vs. CON, and 1.47, for BII vs. CON). Photographs of spiders/bugs were rated as being more arousing by spider-fearful than by BII participants, *t*(31) = 2.60, *p* < .05, *d* = 0.90, but not than by the SOC group, *t*(32) = 1.53, *p* > .1. Blood pictures were rated as being more arousing in the BII than in the SA group, *t*(31) = 3.39, *p* < .01, *d* = 1.19, but not than in the SOC group, *t*(31) = 1.75, *p* = .091, *d* = 0.62. Pictures of social exposure and angry faces were rated as being more arousing in the SOC than in the SA group, *t*s(32) > 2.2, *p*s < .05, ds = 0.8, but not than in the BII group, *t*s < 1, *p*s > .1. Pictures of social exposure and angry faces were assessed as being similarly arousing in the social fear group, *t*(16) < 1, *p* > .1.

##### Valence ratings

**Fig. 9:**
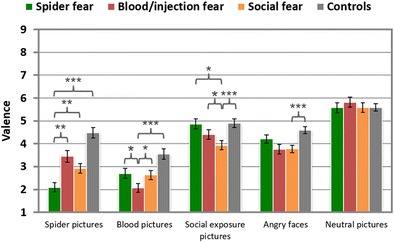
Mean valence ratings of different phobic groups in response to specific stimulus categories. *SEM*s are marked by black lines. ^***^ *p* < .001, ^**^ *p* < .01, ^*^ *p* < .05

The fear groups evaluated their target pictures as being more unpleasant than did control participants, *t*s ≥ 3.9, *p*s < .001 (see Table 4 and Fig. 9). These effects were very strong for all fear picture categories, ranging from *d* = 2.8 for photographs of spiders/bugs (SA > CON), to *d* = 2.0 for blood/injection pictures (BII > CON), *d* = 1.61 for angry faces, and *d* = 1.39 for social-exposure scenes (SOC > CON). No differences in valence ratings for neutral pictures were observed between the control and the three fear groups, *t*s < 1, *p*s > .1. Photographs of spiders/bugs were experienced as being more unpleasant in the SA than in the BII and SOC groups, *t*s > 2.8, *p*s < .01 (*d*s = 1.43 [SA vs. BII] and 0.98 [SA vs. SOC]). Blood pictures were assessed as being more unpleasant in the BII fearful than in the other fearful individuals, *t*s > 2.4, *p*s < .05, *d*s = 0.9. Social-exposure scenes were found to be more unpleasant in the SOC group, *t*s > 2, *p*s < .05 (*d*s = 1.28 [SOC vs. SA] and 0.71 [SOC vs. BII]). Pictures of angry faces were rated with similar valences in the SOC group versus the other fear groups, *t*s < 1.8, *p*s ≥ .1. In the social fear group, similar valence ratings were found for pictures of social exposure and angry faces, *t*(16) = 1.68, *p* > .1.

As expected, fear, arousal, and valence ratings for the social-exposure and angry-face categories correlated with LSAS anxiety subscale scores, *r*s > .3, *p*s < .01. Similarly, fear, arousal, and valence ratings for the blood/injection/injury pictures were positively correlated with MQ scores, *r*s > .6, *p*s < .001, and the assessments made for the spider/bugs category correlated with SPQ scores, *r*s > .5, *p*s < .001.

### Discussion

In Study 2, the extended set of fear-relevant and neutral pictures was assessed by spider, blood/injection, and social-fearful groups, as well as by nonfearful individuals, selected on the basis of fear-specific questionnaire scores. The results showed that fear-relevant images were assessed as being more arousing, fear-evoking, and unpleasant in the three fear groups than in the controls. As in the first study, these groups were also emotionally more affected by the neutral pictorial materials. Moreover, the three fear groups differed with regard to their fear-relevant picture ratings. This differentiation was fairly clear for the spider fear group, in which spider/bug pictures induced stronger negative emotions, and other fear-relevant materials weaker negative emotions, than in other fearful individuals. Blood/injection pictures also tended to be rated as more negative by the blood/injection than by the other fear groups. Differences in the social fear categories were much smaller for the social-exposure pictures, and were not significant for images of angry faces, when the social and blood/injection groups were compared.

Addressing the weaknesses of the original set rated in Study 1, additional spider and neutral pictures were included in the second study. The subset of spider-phobia-relevant photographs was observed to satisfactorily differentiate between spider-fearful and other participants (effect sizes were higher than in the first study). Contrary to our expectations, we still observed greater fear/arousal ratings for the neutral pictures in fearful than in nonfearful individuals. This effect was observed to be even greater in the second than in the first study, despite our efforts to reduce hypothesized aversive context effects by increasing the number of neutral pictures. Importantly, group differences also seemed more pronounced for the other rating materials in the second than in the first study. We suggest that this effect might have resulted from using a more clinically valid way to select the participants in the second study. The present ratings replicated the results of Study 1, showing that the social-exposure pictures elicited greater negative emotional experience in the social-fearful than in the other participants. Also, BII-fearful participants rated these photographs as being less unpleasant and fear-evoking (but not less arousing) than did the social fear group. Interestingly, these two groups were quite similar in their ratings for angry faces, which is a further indication that BII participants are emotionally involved when confronted with some forms of aggression.

## General discussion

The present work reports two separate studies performed in order to develop and validate a set of fear-inducing pictures (SFIP) intended to be employed in various research investigating blood/injection, spider, and social fearful individuals. In order to achieve this goal we collected a large number of high-quality (resolution of 768 × 1,024 or 1,024 × 768) phobia-relevant and neutral photographs that were later rated by social, spider and blood/injection fearful as well as nonfearful control individuals. The results indicate that the phobia-relevant photographs included in the SFIP induce increased fear and arousal responses in spider, blood/injection and social fearful individuals.

As expected, the final SFIP subsets of spiders/bugs and blood/injection photographs were generally assessed as most arousing, fear-evoking and unpleasant. At the same time, these pictures were shown to successfully discriminate between the appropriate fear group and other fearful as well as nonfearful individuals. Specifically, when compared to other participants spider fearful individuals reported greater negative emotions for pictures depicting spiders and bugs. Also, the blood/injection group tended to evaluate photographs including blood and injection scenes as more arousing, fear-evoking and unpleasant than other participants. Supporting the between-group analyses blood/injection/injury pictures ratings were strongly correlated with Mutilation Questionnaire scores and the assessments made for the spider/bugs category correlated with SPQ scores.

In addition to the findings obtained for specific-phobia photographs, pictures depicting social-exposure scenes were overall assessed with greater fear and arousal ratings and were found to be more unpleasant than neutral cues. Moreover, the assessments of social-exposure scenes were strongly correlated with the anxiety subscale scores of the LSAS and indicated that these scenes evoke greater negative emotions in social fearful than other individuals. Our data also demonstrated that these pictures induce similar (or even greater) fear and arousal responses in the social fear group when compared to pictures depicting angry faces. The latter category has been proved to be useful in many previous social phobia studies and was included in the present study to validate the new social phobia picture subset (see Brühl et al., 2014, for a review). Our findings indicate that social-exposure photographs can be employed to study pathological mechanisms in social phobia. As a caveat, it has to be noted that the two social-phobia-relevant picture categories were rated as being less unpleasant and evoked smaller levels of arousal and fear when compared to the spider/bug and blood/injection picture category. The lower subjective emotionality assessments for the social phobia subset may be partly related to clinical differences between the specific and the social phobia: when compared to specific phobics, social phobics have a tendency to use emotional regulation strategies during the exposure to their feared objects/situations.

Apart from the expected phobia-specific assessments, fearful individuals showed higher arousal, fear and lower valence ratings for neutral SFIP photographs. This effect might indicate the generalization of a negative emotional experience in fearful individuals exposed to a fear-relevant context. Supporting this assumption, several previous animal and human studies reported strong sensitization effects in a fear-relevant context (Bublatzky & Schupp, 2012; Michalowski et al., 2015; Rau et al., 2005). For example, neuroimaging data showed that phobic participants exposed to a context in which their feared pictures are likely to occur respond with greater brain response to fearful but also to fear-irrelevant pictures (Kolassa et al., 2006; Michalowski et al., 2009; Straube et al., 2007; Weymar, Keil, & Hamm, 2014). In the present study, similar sensitization effects might have resulted in an increased subjective experience of arousal and fear in fearful individuals leading to an overestimation of emotional experience for neutral pictures. This effect was observed even though in Study 2 the number of neutral pictures was about six times higher than the number of photographs used in Study 1.

The generalizability of the present findings might be limited to the included sample. Our findings were obtained with subclinical participants and cannot be directly transferred to anxiety patients. Moreover, the study included only students and mainly women. However, phobias are more prevalent among women, and we have no reason to suspect that men or nonstudents would differ in terms of the effects observed in our study. The sample included in Study 2 was relatively small, and further studies with larger samples are required to confirm the normative rating data presented for the SFIP. It is also important to note that the adopted recruitment criteria (see methods section) might have resulted in a greater interdependency of the three fear groups. Even though such interdependency is not surprising considering a high co-occurrence of different phobias (LeBeau et al., 2010), it has most likely led to a reduced rating differentiation between the three fear groups. Moreover, some inconsistencies were observed in the present study between fear, arousal and valence ratings suggesting that fear-relevant SFIP pictures might have also addressed emotions other than fear. For example, fear-relevant pictures are sometimes producing disgust instead of fear (see, e.g., Olatunji, Cisler, McKay, & Phillips, 2010). It is also possible that the picture presentation time, lasting 3 s in our Study 2, might have been too short to let our participants easily perceive and define their basic emotional experience. These factors might have affected our findings leading to the reduction of fear ratings. Assessing the SFIP pictures with regard to other basic emotions and extending the duration of picture presentation in future studies may help to understand the inconsistencies mentioned above.

We also think that the SFIP might be further expanded. For example, in the future it might be useful to complete the SFIP with faces expressing other negative and also positive emotions, since there is evidence for increased processing of negative and positive facial expressions in high socially anxious people (Chen, Ehlers, Clark, & Mansell, 2002; Mansell, Clark, Ehlers, & Chen, 1999; Wieser, Pauli, Weyers, Alpers, & Mühlberger, 2009), an effect that might be associated with the increased fear of negative and positive evaluations that is postulated for social anxiety (Weeks, Heimberg, Rodebaugh, & Norton, 2008). Moreover, the SFIP may be supplemented with additional spider pictures in the future. The actual spider-fear-relevant picture subset consists of photographs of spiders (75 %) and bugs (25 %). Including photographs of bugs might reduce the intensity of fear-specific effects in spider-fearful individuals. On the other hand, the results from the present study revealed that this composition of the spider-fear-relevant picture subset was still sufficient to elicit increased fear and arousal responses in this group. Finally, it might be desirable to check the validity of the SFIP using more objective neuroimaging or peripheral physiological measures.

## Footnotes

1 The between-group effects were unstable across different subcategories of small animals. For example, no differences between the three fear groups were observed for the fear and arousal ratings of bats, mice, and rats: *t*s < 1.5, n.s. Also, the small-animal fear group did not differ from the BII fear group in their fear and arousal ratings for snakes, or from the social fear group in their fear and arousal ratings for flying insects: *t*s < 1.7, n.s.
